# Pregnant womens’ concerns when invited to a randomized trial: a qualitative case control study

**DOI:** 10.1186/s12884-015-0641-x

**Published:** 2015-09-04

**Authors:** Katrien Oude Rengerink, Sabine Logtenberg, Lotty Hooft, Patrick M. Bossuyt, Ben Willem Mol

**Affiliations:** Academic Medical Center, Department of Obstetrics and Gynaecology, Meibergdreef 9, 1105 AZ Amsterdam, The Netherlands; Onze Lieve Vrouwen Gasthuis, Department of Obstetrics and Gynaecology, Oosterpark 9, 1091 AC Amsterdam, The Netherlands; Department of Epidemiology, University Medical Center Utrecht, PO Box 85500, 3508 GA Utrecht, The Netherlands; Academic Medical Center, Department of Clinical Epidemiology, Biostatistics and Bioinformatics, Amsterdam, The Netherlands; The Robinson Institute, School of Paediatrics and Reproductive Health, University of Adelaide, Adelaide, Australia

## Abstract

**Background:**

Pregnant women were excluded from clinical trials until the 1990s, but the Food and Drug Administration nowadays allows - and even encourages - responsible inclusion of pregnant women in trials with adequate safety monitoring. Still, randomized trials in pregnant women face specific enrolment challenges. Previous studies have focused on barriers to trial participation in studies that had failed to recruit sufficient participants. Our aim was to identify barriers and motivators for participation in a range of clinical trials being conducted in the Netherlands, regardless of recruitment performance.

**Methods:**

We performed a qualitative case control study in women who had been asked in 2010 to participate in one of eight clinical trials during pregnancy or shortly after giving birth. Both participants and non-participants of these clinical trials were invited for a face-to-face interview that addressed motives for participation and non-participation. We started the interview in an open fashion, asking the women for their main motive for participation or non-participation. When no new information emerged in this open part, we continued with a semi-structured interview, guided by a topic list. Transcripts of the interviews were analysed using a constant-comparative approach. Two researchers identified barriers and facilitators for participation, conjoined into main themes.

**Results:**

Of 28 women invited for the interview, 21 agreed to be interviewed (12 participants and 9 non-participants). For 5 of the 12 participants, contribution to scientific research was their main motive, while 5 had participated because the intervention seemed favorable and was not available outside the trial. Key motives for non-participation (n = 9) were a negative association or a dislike of the intervention, either because it might do harm (n = 6) or for practical reasons (n = 3). Combining the open and topic list guided interviews we constructed seven main themes that influence the pregnant women’s decision to participate: external influence, research and healthcare, perception own situation, study design, intervention, information and counselling, and uncertainty.

**Conclusions:**

Among seven main themes that influence pregnant women’s decision to participate, uncertainty about scientific research or the intervention was reported to be of considerable importance. Measures should be taken to habituate pregnant women more to scientific research, and further evaluation of opt-out consent deserves attention.

**Electronic supplementary material:**

The online version of this article (doi:10.1186/s12884-015-0641-x) contains supplementary material, which is available to authorized users.

## Background

Until the 1990s, pregnant women were often excluded from clinical trials for their own protection [1]. However, in general pregnancy does not prevent a women from acquiring a disease or cure a women from a disease. Pregnant women may even be more severely affected, for example by infectious diseases [2]. Paradoxically, the efforts to protect the fetus from research-related risks by excluding pregnant women from research places both at risk from unstudied interventions [3, 4].

In the United States approximately 2 in 3 pregnant women are given prescription medication during pregnancy [2]. These prescriptions are often based on limited evidence on safety or effectiveness, as results of studies in non-pregnant women may not always apply to pregnant women. The Food and Drug Administration nowadays allows – and even encourages – responsible inclusion of pregnant women in drug trials with adequate safety monitoring [5].

Randomized trials in pregnant women still face specific enrolment challenges. Such studies are unique, since two patients are involved: the mother and her unborn fetus. A woman may refuse treatment for herself if she feels this could harm her baby, or she may feel bound to accept interventions that might benefit the fetus. Additionally, the father’s feelings may also influence decision-making about trial participation [6].

Tooher and colleagues have presented a narrative review on factors influencing recruitment for maternal and perinatal trials in which they identified four participant factors that influence recruitment: understanding risk, recruitment process and procedures, participants’ understanding of the research process and methodological issues, and patient characteristics [7]. Their conclusions were based on a limited number of studies on maternal and perinatal trials, often selected because recruitment was problematic. It is therefore uncertain to what extent these results also apply to other studies.

We performed a qualitative study to identify the main barriers and motivators for enrolment in obstetrical trials in the Netherlands, regardless of recruitment performance.

## Methods

### Design

We performed a qualitative case–control study. Women invited to one of eight clinical trials during or shortly after pregnancy were invited for a face-to-face interview about their main motives in accepting or declining the invitation to participate. This study is part of the IMPACT project, in which enrolment of patients in trials is studied at different levels [8]. Our study did not require formal approval of an ethics committee, according to Dutch law, as confirmed by the ethics committee of the Academic Medical Center and the Onze Lieve Vrouwe Gasthuis in Amsterdam. Written informed consent was obtained for all face to face interviews, and verbal consent was obtained for telephone interviews.

### Selection of trials and interviewees

We invited 28 women who had been invited for a clinical trial in obstetrics up to three months prior to the interview . We selected in a 1:1 ratio women who had accepted and women who had declined enrolment. Women were selected from eight multicenter studies running in the Consortium for Women’s health and Reproductivity that recruited patients between February and June 2010: Allo [9], Apostel I [10], Apostel II [11], Chips [12], WOMB [13], Ppromexil [14], Hypitat2 [15], and ProTwin trial [16] (Table 1).

**Table 1:** Overview of the eight trials from which patients were selected for an interview

Trial acronym^a^	Research question	Treatment arms	Eligible women
Allo [9]	Does antenatal allopurinol administration reduce hypoxic-ischaemic encephalopathy in neonates exposed to intra-uterine asphyxia?	Allopurinol or placebo, antenatal administered to the mother	Women at term in whom the fetus is suspected of intra-uterine asphyxia
Apostel I [10]	Is testing for fibronectin a cost-effective strategy that prevents unnecessary treatment in women with threatened preterm labour?	Tocolytics (nifedipine) or placebo	Patients with symptoms of preterm labour, and a negative fibronectin test and a cervical length between 10–30 mm
Apostel II [11]	Does sustained tocolysis in women with threatened preterm labour reduce neonatal morbidity?	Nifedipine or placebo for 12 days	Women between 24 to 31^+6^ weeks pregnant who have been treated with tocolysis and steroids for preterm birth for 48 h
CHIPS [12]	Is there a difference on pregnancy loss or NICU admission between less tight and tight control of blood pressure in women with non-severe non-proteinuric pre-existting hypertension or gestational hypertension remote from term?	‘less tight’ dBP control or ‘tight’ dBP control	Women with non-severe non-proteinuric pre-existing hypertension or gestational hypertension remote from term
Hypitat II [15]	What is the effectiveness and efficiency of induction of labour in women with pregnancy induced hypertension or mild preeclampsia with a gestational age of 34–37 weeks of pregnancy, as compared to expectant management under regular monitoring?	Induction of labor or expectant management under regular monitoring	Women with pregnancy induced hypertension or mild preeclampsia with a gestational age of 34–37 weeks of gestation
Ppromexil [14]	What is the effectiveness and cost-effectiveness of induction of labor after PPROM between 34 and 37 weeks gestation compared to expectant monitoring.	Induction of labor or expectant monitoring	Pregnant women with preterm premature rupture of membranes between 34 + 0/7 weeks to 37 weeks of gestation
ProTWIN [16]	Is prophylactic use of a cervical pessary effective in the prevention of preterm delivery and the neonatal mortality and morbidity resulting from preterm delivery in multiple pregnancy?	Pessary or no treatment	All women presenting with a multiple pregnancy between 12–20 weeks of gestation
WOMB [13]	What is the effect of RBC transfusion on health related quality of life?	RBC transfusion or no intervention	Women with PPH or a decrease in Hb, 12 to 24 h after delivery or caesarean section.

^a^More information about these studies can be found at: www.studies-obsgyn.nl

We started by inviting the women most recently invited for a trial, and thereafter selected women less recently invited consecutively (up to 3 months prior to the interview). Women were only eligible if they were still pregnant or their baby was born alive and if they could speak Dutch well enough to participate in the interview without an interpreter. We first sent an invitation letter on behalf of the treating gynaecologist and the interviewer introducing the study and the purpose of the interview. The letter indicated that participation or non-participation in the interview was completely voluntary and would not influence their relationship with the treating physician or her treatment in any way. We announced to the women that we would try to contact them by phone about a week after having received the letter, to give additional explanation and answer any remaining questions. At least four attempts to reach the women by phone were made. If she indicated she was not interested and did not want any additional information her wishes were respected, and reminders were not sent.

### The interview

The interview was conducted face-to-face, unless the respondent explicitly requested a telephone interview, or when the travel time to visit the patient was 2 h or more. The interview took place at the patient’s home or in the hospital, whichever was preferred by the interviewee. An interview in the hospital was proposed as the hospital could be perceived as a location were women feel comfortable talking about trial participation, as they might feel uncomfortable inviting the interviewer to their homes, but it could also be because women or their newborns were still admitted.

We started the interview in an open fashion, by asking the women for their main reason for participating or not participating in the trial. Once no new information emerged from the open questioning, we continued with a semi-structured interview guided by a topic list, to cover all aspects that might have contributed to the decision making process.

The topic list was developed based on a literature review and with input from experienced gynaecologists and midwives (Additional file 1: Appendix 1, Dutch). It included factors related to personal benefit, altruism, knowledge and information about the trial and the trial process, distrust, attitude, organisational aspects and influence of the social environment. If new topics emerged during the interview, they were added to the topic list [17, 18].

All interviews were recorded and transcribed. Participants were asked for verbal consent for audiotaping, after verbal consent was given the audiotape was started. We informed the women that whenever they felt uncomfortable during the interview, they were permitted to stop the interview at any time, even for no reason. The transcript was sent to the interviewees to confirm correctness and completeness (member check). If the women indicated that the interview was not a good reflection of their motives or they did not want their information to be used any longer, the information was not used. All transcripts were coded using one letter of the alphabet, with the code only known to the interviewer and transcriber (SL and KOR). Transcripts were not shared with their treating physician.

We estimated that an interview with about 10 women in both groups would be needed to reach data saturation, based on previous papers published [8]. We planned to perform two additional interviews when data saturation was reached.

### Analysis

The aim of the analysis was to group the content of the interviews into main themes. Analysis was performed according to the taxonomy of Strauss & Corbin (‘create theory out of data’), where one starts with line-by-line open coding of all relevant phrases of barriers or motivators for participation (open coding), using a constant comparison method: newly gathered data are continually compared with previously collected data and their coding, in order to refine the development of theoretical categories [18].

After open coding, the codes were grouped into categories (axial coding), and then into themes (selective coding) [18]. All transcripts were reread and recoded, using the refined coding structure. A fragment was placed into all relevant categories. Two researchers (SL and KOR) independently analysed the first seven interviews, thereafter one researcher marked barriers and facilitators, which was checked by a second researcher. Discussion was resolved by consensus if needed. For the purpose of this article direct quotes from the interview were translated by a professional translator, after analyses had been finished.

## Results

### Interviewees

Of the 28 identified women, four women could not be reached by phone, two non-participants declined an interview, and one woman initially consented but was admitted to hospital for emergency care. Her interview was cancelled. Twenty-one interviews were performed (12 with trial participants and 9 with non-participants), of which 17 were face-to-face and 4 were by phone. The interview took on average about half an hour. After transcription of the recorded interview, 20 interviewees approved its content, while one woman did not respond. Characteristics of the interviewees are shown in Table 2. Five women had been invited to this study more than three months after being invited to a trial, mostly due to incomplete registration of non-participants. All respondents stated they remembered the situation that was discussed in the interview very well, which we confirmed during the interviews. We considered data saturation reached, as no new motives were mentioned during the last two interviews. As we sampled participants from different studies, we defined data saturation as no new motive emerging over all studies. For example, we considered ‘distrust in the effectiveness of a new intervention’ as a motive, whether this was a pessary in the ProTwin trial or iron tablets in the Womb study.

**Table 2:** Characteristics of the women included

Code	Ethnicity	Level of education	Age	Study	Parity^a^
Participants					
J	Dutch	Intermediate/low	30-34	Allo	Nulliparous
L	Dutch	Higher education	35-39	Allo	Parous
M	Dutch	Higher education	30-34	Allo	Parous
N	Dutch	Higher education	25-29	Apostel I	Parous
P	Dutch	Higher education	25-29	Ppromexil	Nulliparous
Q	Dutch	Intermediate/low	25-29	Ppromexil	Nulliparous
T	Dutch	Higher education	30-34	Hypitat II	Parous
U	Dutch	Higher education	35-39	ProTWIN	Nulliparous
V	Non-Dutch	Higher education	35-39	Hypitat II	Parous
W	Dutch	Higher education	35-39	Apostel II	Parous
Y	Non-Dutch	Higher education	35-39	CHIPS	Parous
Z	Non-Dutch	Higher education	25-29	WOMB	Parous
Non-participants					
A	Dutch	Higher education	35-39	WOMB	Parous
B	Dutch	Unknown	25-29	ProTWIN	Nulliparous
C	Dutch	Higher education	30-34	ProTWIN	Parous
D	Non-Dutch	Higher education	30-34	ProTWIN	Parous
E	Non-Dutch	Higher education	35-39	Hypitat II	Parous
G	Dutch	Higher education	25-29	Apostel II	Nulliparous
I	Dutch	Higher education	20-24	ProTWIN	Nulliparous
K	Dutch	Intermediate/low	20-24	Ppromexil	Parous
O	Dutch	Intermediate/low	20-24	Hypitat II	Nulliparous

^a^Parity was registered at the time of the interview

### Main motive for trial participation or non-participation

The main motives for trial participation as mentioned by the women are shown in Table 3. Contribution to scientific research was for 5 of the 12 participants their main motive for participation in the trial, sometimes conditional upon other motives (J, L, T, U, Z). Five participants mentioned to have participated because the intervention seemed favorable and was not available outside the trial (M, P, Q, V, W). One woman thought an extra test could only have positive effects, as ‘there is no harm in trying’ (N). For one women the reason was not very clear, she most probably meant to be better informed about her medical condition (Y). Key reported motives to participation for the 9 non-participants were a negative association or dislike of one of the interventions, either because it might do harm (C, D, E, G, I, K) or for practical reasons associated with the intervention (A, B, O). “Women also described first-time pregnancy or being in an exceptional situation as playing a role.”, or she was already in an exceptional situation, like a twin pregnancy or a pregnancy after intrauterine insemination.

**Table 3:** Main motives for participation or non-participation as answered to the starting (open) question

Code	Citation
J	“I don’t think research is ever actually bad, and this is not a study where they do real experiments, so it’s always good to learn from it for someone else.”
L	“Actually, in our first pregnancy our daughter was in foetal distress and so we had to have a caesarean section. This might have been an option then, too, as it has something to do with foetal distress, then administering this. And my husband actually asked more questions: does it have drawbacks for the child? No? Then we’ll join, because the study is necessary.
	There’s also my medical background. I’ve worked on maternity wards, too. When you work in medicine, you’re open to innovation and new techniques.”
T	“Two things, actually. In my first pregnancy I had pre-eclampsia, so I was very well aware what the consequences might be for me, and then also for the child… Personally, I support the aims of the study, to let you have your baby from 34 weeks onwards, because the risks do not outweigh for mother and child, so to speak. Second, I’ve been coming to a teaching hospital for years, for other treatments as well, and I believe very much in the academic side. I believe in development and trying new things. And, well, research is part of that because if you never do any studies, you can never do anything new.”
U	“Well, originally I was invited to take part in a study about the pessary, a study of twins. I thought: seems good to me, I have a twin sister myself and I used ICSI to conceive, so there were also people who took part in this kind of study for me. That’s how I’m pregnant now.”
Z	“Well, first, it did really apply to me and there was the choice between taking blood or iron. Otherwise it would have been iron, whatever. So I thought, let’s see what happens with this. And I was in the blood group. Looking back, I’m very happy with it. And, as I just said, I often do studies myself. So then you know better how important it is, that you need to recruit people, so erh, actually that’s the only reason.”
M	”The most decisive factor, of course, is that the consequences of oxygen deprivation are pretty severe. If you could reduce that in some way, by taking a particular drug, then I’d choose it. Yes, yes, good. And because the drug was already being used for other things – OK, so it hadn’t yet been fully tested for oxygen deprivation – then it shouldn’t have any bad effects. You assume that it can only be beneficial. And so then I think, like, that’s something I want to take part in.”
P	“OK, well, that was mainly due to the fact that there was a chance that my labour would be induced, otherwise I’d have to wait until 37 weeks come what may… The contribution to research as well, of course, I thought that was a good cause, but it wasn’t the most important. I thought: I’m going for immediate induction. I couldn’t imagine having to stay in hospital for five days, not allowed to do anything, so I thought, like, let it come now.”
Q	“First of all, I don’t see myself lying here for another five weeks. And pretty soon after that the realisation that you’re already open down there, with a risk of infection for yourself and for the baby. And yes, in Enschede the doctors also said it was viable enough, so that was for us a reason to take part.”
V	“That once the baby was out my high blood pressure would be gone. That’s what I thought, that was about it. But on the other hand, I was a bit scared. Will I have him earlier – that was at 36 weeks – so it was a bit of a dilemma deciding what would be best. Then she explained to me: the earlier the baby’s out, the better it should be for mother and child. So that was actually the reason why I said I’d do it.”
W	“That was because I hoped it would be better for the baby, although I still had an uneasy feeling about it. That was because nobody could say what the potential adverse effects were. Yes, I kept on feeling uneasy about it.”
	“And I had something like, in my case it can only be positive, because I mean, the test would indicate whether the chance was very high that you would deliver very soon, or that it could take a while. So, I really felt like, I felt that I ran little risk, because if the test would show that you would fall into the test group, than you would get either a placebo or tocolytics.”
N	“And I thought something like, in my case it can only be positive because, I mean, the test would indicate whether the chance was very high that you would deliver very soon, or that it could take a while. So I really felt like I wasn’t running much risk, because if the test showed that you fell into the test group, then you would get either a placebo or tocolytics.”
Y	“Then you know how and what.”
C	“Well, there were several reasons actually. When your colleague started talking about it, when I had an appointment about it, I thought: ‘Oh my God, no, not a pessary! Because I had a friend who was admitted to hospital because a pessary [not in pregnancy] had caused a lot of bleeding. So that’s what I told her [the colleague]: that that had been a life threatening situation. So I had a feeling of, like, if I think now about pregnancy and a pessary, it doesn’t make me very happy.”
D	“For me it was pretty clear, actually. Once I was here I thought, like, just let Mother Nature do her work. I’m pretty religious [Muslim], so perhaps there’s a reason why those children are born early. I believe in God, you know. I think, like, fate decides. If those children want to be born earlier, then so be it. If not, then not. That was my thinking. I was scared, too. What if I take part and something happens to me, a bit of blood loss – or a lot – or something happens to the babies.”
E	“She [baby] was four weeks early and that blood pressure kept on rising. They just couldn’t get it down. I’d already been lying there for a month and I’d had enough. You want something to happen. Then they asked me: do you want to take part in this study? Because there’d come a point when the doctors were saying, we don’t know any more, either. So I thought, well, if they don’t know, who does? I had to make my own choice. … And then I thought, actually I can better prolong it for a while, to see how long it takes. Because if I had decided to take part, you don’t know whether you’ll be induced or not. That’s not certain, either. So then the disappointment can be huge. That was when I decided not to do it, to see how long we could prolong it.”
G	“I had a very tough pregnancy, with a lot of bleeding, and in fact the whole nine months were entirely uncertain… I was given those lung development injections and I tocolytics, after which I couldn’t feel my baby at all… When they asked if I wanted stay on the tocolytics, I linked them a bit with that so I thought, like, no – because I wanted to feel my baby again as soon as possible, to regain a bit of the certainty that everything was alright. So for me, that was the most important reason.”
I	“At first I was really inclined to participate, because a lot of people close to me said, just say it works. Your babies will stay inside longer. But personally, I had the feeling that everything was going very well, that it all, yes… And I do react quite strongly to things, to jewellery or a piercing or something. So I think, if something’s going to be stuck inside my body, it might react really badly. If nothing’s wrong and I do that… I found that a bit scary. And then there’s the fact that you couldn’t choose which group you were in. That’s logical with a study, but that’s why I didn’t do it in the end.”
K	“Well, it’s not without a reason that they tell you that you’re officially allowed to deliver from 37 weeks on, so yes, I thought it was a risk being induced at 34 weeks. Because the doctors don’t just say: from 37 weeks the doctors will induce you automatically and they’re doing a study and I didn’t want to be a guinea pig. If something then goes wrong …”
A	“I would happily have taken part if I could have opted for iron tablets, but that choice wasn’t available. You have to participate blind, and then I don’t know who decides. I don’t know how that works, but someone else decides for you which of the two you are going to do. What’s also complicated: I didn’t want a blood transfusion. I was lying there on a drip and I had a catheter, and then I thought that with iron tablets I could go home and otherwise I would have to stay even longer.”
B	“I’m at the AMC. That’s a teaching hospital and they do all kinds of research there. I would have to come in more often – it was all about a pessary against premature birth – and I would have to come in more often to measure it up and for ultrasound. I did seriously consider it, but those extra visits… If was in pain, for example, or it wasn’t convenient. And I’d just heard I was pregnant with twins sharing an amniotic sac, which is a very rare situation – you have a lot information coming at you.”
O	[Unplanned pregnancy] “Yes, and everything suddenly went so fast. Then I really thought, like, well, I don’t have to be induced tomorrow. That… the chance was 50%, and I didn’t need that. No, I feel it’s all gone too fast. Because you’re… No… After three weeks attending the hospital, I was admitted. I’d never been in hospital before and… Yes, yes, I was homesick. Yes. But, I didn’t think, like, whip him out tomorrow. Really, that just wasn’t what I wanted. That was simply too fast for me. I couldn’t take it all in.”

### Themes identified as related to the decision on trial participation

During the open coding we identified 47 sub-codes, based on phrases relating to barriers and facilitators. These were grouped into 13 categories, and further grouped into seven main themes (Table 4), discussed below.

**Table 4:** Seven main themes that influences trial participation

Theme	Sub codes
External influence	▪ Concern from social environment
	▪ Trust in the health professional
	▪ Feeling of disappointing the health professional
Research and healthcare	▪ Familiarity with scientific research
	▪ Willingness to contribute to research
	▪ Feeling of participating in an experiment
Perception own situation	▪ Perception own situation and medical history
	▪ Feeling very eligible or very ineligible for scientific research
Study design	▪ Randomization
	▪ Blinding
	▪ Placebo
	▪ Additional efforts
	▪ Insurance medical research
Intervention	▪ Intervention
	▪ Natural course
Information and counseling	▪ Written information
	▪ Counseling: information and timing, atmosphere
	▪ Time for consideration on participation
Uncertainty	▪ Fear
	▪ Stress
	▪ Doubt
	▪ Physician does not know what is best

### A. External influence

Women indicated that they discussed the invitation to enroll in a trial with their partner, where the partners opinion influenced the decision on whether to participate or not. This influence could be either positive or negative, giving the women more confidence to decide to participate or withholding her from participation if the partner perceived more risks of participation. In two cases the woman and her partner disagreed. Women indicated that opinions of persons other than their partner were not very influential.

“I did discuss it with my husband. Myself, I already thought, like, I’m fine with it. My attitude did depend on what my husband would say, too, but he said something like: that’s good. So we agreed. Interviewer: “And if he [your partner] hadn’t agreed?” Participant: “Then I wouldn’t have done it.” [Participant Allo trial]

Women indicated they had decided on participation without consulting their gynecologist, however when the gynecologist was contacted, his or her opinion was in most cases influential. All respondents felt free to make their own decision, without feeling pressure from anyone to participate.

### B. (Contribution to) research and healthcare

Contribution to scientific research was a reason for participation, as they were convinced about its importance.

“And I also looked at it like this: these are studies for the future, and after all I have a daughter and you never know. In that case I’m the kind of person to take part in things for other people, so that it’s better in the future than it is now, for example. What other people have done in the past, I’m making use of now.” [Participant Hypitat II trial]

Interviewees who had declined participation judged scientific research also important, but other themes outweighed this importance. One participant suggested to improve publicity on clinical trials and research in pregnancy:

“Maybe they should be a bit more up-front about saying, if people are pregnant anyway, that there are studies that people can take part it. Maybe that it’s passed on to people in some way or another as soon as they fall pregnant, so that you know about it. I didn’t think about it. I’ve never experienced this before. I think that would cause less stress. Say you get leaflets in advance, “scientific research for pregnant women”, and you’ve read them. Then you know already that it might come up. You can already think about it.” [Participant Ppromexil study]

### C. Perception own situation

The perception of one’s own situation appeared influential in the decision on participation: women considered themselves either very eligible or not at all eligible for scientific research, sometimes taking into account their current complicated pregnancy, medical history or their own nature.

“There are people who do that [participate in trials], and that’s all very wonderful and good, but I’m not that kind of person. You know, no playing about my body. Maybe if it was just one [baby], but now it’s already scary and if there’s going to be all that fiddling with your body, then give me the natural way.” [Non-participant ProTwin]

Intuitional or emotional elements were also reported. When asked whether their decision was rational, some women answered they trusted their feelings, or were inclined to participate but did not participate because it did not feel good.

### D. Study design

Randomization was perceived negative and resulted in uncertainty. Women could not explain (in any way) why randomization was used, or could be used. This seemed based on a combination of not knowing why randomization could or should be used, and not trusting that there is really equipoise and the doctor does not know what is best for them. This seemed based on a combination of not knowing why randomization could or should be used, and not trusting that there is really equipoise and the doctor does not know what is best for them. In the Netherlands, when explaining randomization as ‘loten’ a Dutch word meaning drawing straws that has the connotation of faith or destiny, this was perceived negative, while explaining randomization by means of a computer selecting a group for the participant, this was considered more positive. Therefore Dutch doctors should avoid this.” However this lack of knowledge was not necessarily a barrier for participation.

“Because if I had decided to take part, then are you going to be induced or not? That’s also a doubt. So then the disappointment is huge. No, the uncertainty; [if you decide for yourself,] you go in and you know where you’re going.” [Non-participant HYPITAT II]

### E. Intervention

Participants mentioned the potential therapeutic benefit of the intervention as a reason for participation.

“Well, yes, if they stay inside longer in that case, than that’s an advantage for me, too. Actually, that was my only reason for taking part. But I did need to be convinced that there wouldn’t be any drawbacks if they didn’t stay inside so long because of it.” [Participant ProTwin trial]

Other women disliked an interventional (“active”) strategy, and rather preferred the natural course, or were more focused on potential (unknown) negative effects. They mentioned that the risk associated with a natural course is one you do not choose for. It is already present, contrary to the risk of an intervention or the risk of trial participation, which result from the women’s choice. All non-participants stated that a negative association with the intervention or a negative effect of intervention played a role, as discussed under the main motivations.

“To me it was already pretty clear, actually. Once I was here I thought, like, just let Mother Nature do her work. I’m not going to mess around with with something if nature has decided that’s the way it is. I let it be, you know.” [Non-participant ProTwin trial]

### F. Information and counseling

Women considered the information adequate, but indicated that the counseling was done very hastily. A no rush atmosphere, where counseling was often done by a research nurse or midwife, with sufficient time to discuss questions, was viewed as positive.

“But maybe with hindsight I think it matters a lot: the person who comes to tell you [about the study]. If someone like Ms J [the research midwife] had come to my bed at the beginning and taken her time over it, then maybe I would’ve thought differently than when you have a doctor perched on the window-sill, only just not looking at her watch, saying I’m just popping in for five minutes and then I’m gone and you have to make a decision. Because you have to join the study at two o’clock this afternoon, otherwise it’s too late. Yes, that feels different and the results are different. Perhaps even when it comes to taking part in the research.” [Participant Apostel II]

One women said she had received unclear and incomplete written information, but nonetheless she participated.

“These are the same tocolytics you normally get, they’re no different. It’s not a new drug, but I only realised that later. It wasn’t made clear at the time I had to decide. It looked like it was a new drug, or a new way of seeing whether it can be kept inside longer if your waters break early, and what harm that could do to the child or the mother. In other words, in the future, when people come in with waters that have broken early, can we give them these pills with confidence? That was my feeling about the study, actually, and when I had to say yes I didn’t know that it was the same drug and what harmful effects it might have. If that was all explained very clearly then, including the harmful effects it might have – that’s the research, of course – then I’d join more easily. If they can say that the only harmful effect is that he’ll a bit smaller or a bit bigger, or a bit more to the left or a bit more to the right… But if you don’t know that, then it’s pretty difficult. It’s a whole ethical issue.” [Participant Apostel II]

Women felt the time to consider participation was adequate, or they understood why the time for consideration was short, except one woman. Some women declined participation because there was an overwhelming amount of new information, or timing was not right.

### G. Uncertainty

The theme uncertainty emerged in interviews of both participants and non-participants. Non-participants explicitly mentioned to have declined participation because of feelings of uncertainty. This prevailed over other factors even before they had reached the stage of explicitly weighing the advantages and disadvantages of participation

“No, I didn’t consider that at all, actually. I didn’t think about it. For me the safety of the baby had to come first. No, I didn’t see any benefit in it. No, they [the doctor/counsellor] didn’t bring up the advantages side and I didn’t ask about it.” [Non-participant Ppromexil trial]

In both groups women indicated that being confronted with an (unexpected) invitation to participate in scientific research was stressful and needed thorough consideration.

“I don’t know if it’s really stressful, but we have talked about it a lot. You know, with my husband and also with a friend of mine in Rotterdam. And I have been very busy with it in my head, but whether it’s caused physical stress? I don’t know. Yes, I have thought a lot about it, because you can never be right. If he’d been born and there was something wrong and I hadn’t taken part in the study, then I would’ve thought: if only I had taken part in the study. And if he’d been born and I had taken part and there was something wrong, then I’d have thought: if only I hadn’t taken part in the study.” [Participant Apostel II]

Some women were really surprised when confronted with the fact that ‘2010 state of the art health professionals’ do not always know what treatment is best.

“They [the doctors] also said, ‘We think it’s a very silly thing to say, and very strange for you, but we have to be honest: we don’t know. And I was lying all the time, thinking ‘I’ll see what happens’. Until that moment, when I thought, well, actually I felt I’d been left to my fate. You’re not lying there for nothing. They know it, they’ve studied for this. I’m sure they could tell me what direction it’s best to go in, but apparently they can’t. And that’s really tough. They could only give you certain facts. Well, not even that, really, just that research was there for a reason.” [non-participant Hypitat II]

## Discussion

Contributing to scientific research was for many participants their main motive for participation in the trial, while others were motivated to participate because the intervention seemed favorable and was not available outside the trial. Key motives for non-participation were a negative feeling towards the intervention, either because it might do harm, or for practical reasons. We identified seven themes that influenced the decision to participate in a trial. We noted that uncertainty about scientific research or the intervention was of considerable importance.

This study examined a variety of trials, which were not selected based on their recruitment performance. We sampled patients from multiple trials, multiple centers, invited to enroll by various health professionals, in different geographical areas in the Netherlands. The response rate was high. More non-participants declined an interview, however only two of eleven non-participants invited by phone declined to be interviewed. All interviewees stated that the counseling and the decision making process were very well remembered, which was confirmed by the interviewer. Therefore, we think this did not very much influence the reported barriers and motivators. The varying settings of the interview – either at home or in the hospital – could have influenced the results. As we left the choice for the setting to the interviewee, we think the interviewee had chosen the setting where he or she felt most comfortable to talk about their decision. Most interviews were at the woman’s home. We felt the setting did not restrict women to talk openly about their motives for or barriers to trial participation.

A potential limitation of qualitative research is the introduction of bias, as interpretation is an inevitable part of the analysis of the transcripts. We therefore relied on two researchers to examine the transcripts. Another limitation is the relatively small sample, were we included a high rate of women with higher education. However, views of women with lower or intermediate education were represented. Moreover, in the group of participants more women seem to be multiparous and older than in the non-participants group. A possible explanation for this, is because they are more familiar to being pregnant and they already delivered before, and therefore multiparous women more often participated. However, given the small sample we cannot exclude that these differences are due to chance.

We considered the sampling of women over multiple studies as a strong point of our study, improving generalizability, but given the small sample it is difficult to determine the effect of the sampling from the different studies on the reported barriers and facilitators. This has affected the relative importance of the different barriers and motivators, but this study was designed to get an overview of the main aims for participation or non-participation in trials.

We considered data saturation reached, defined as no new information emerging during the last two interviews. However, the interpretation of data being saturated could be argued. As we sampled from different studies, we considered ‘distrust in the intervention’ as a motive, whether this was a pessary in the ProTwin study or iron tablets in the Womb study. We think this was adequate for getting an overview of the reasons to participate or not participate, but adding more studies might lead to more variety within the motive ‘distrust in the intervention’. In order to quantify or to determine the magnitude of the results in the total population, a larger cross-sectional survey in a representative group of women should be done.

The seven themes identified in this study have been reported previously. Kenyon et al. conducted interviews with women who had participated in the ORACLE study, a randomized trial investigating the value of administration of antibiotics during premature labor. They concluded that women gave prominence to the socio-emotional aspects of their interactions with healthcare professionals in making decisions on trial participation. The interviews suggested that the stressful situation (of being asked to participate in a trial) affected their ability to absorb the information. The main motivation for trial participation was the possibility of an improved outcome for the baby. Another important motivation was an opportunity to help others, but this was conditional on there being no risks associated with trial participation [19]. McCann and colleagues introduced the term ‘conditional altruism’, which describes that the willingness to help others initially inclines people to participate in a trial, but is unlikely to actually lead to trial participation unless people also recognize that participation will benefit them personally [20].

Uncertainty due to unfamiliarity with research or research methods was also identified as a theme related to trial participation in pregnant women [6, 21], and in a systematic review not restricted to pregnant women [21]. Unfamiliarity with randomization was a reason for uncertainty; for many patients it remained unclear why randomization is used. Robinson and colleagues investigated lay public’s understanding of equipoise and randomization in hypothetical randomized trials [22]. Even participants who could correctly explain the rationale behind random allocation doubted the possibility of equipoise and saw no benefits of random allocation over the doctor/patient choice. They concluded that, given the extent of disparity between the assumptions underlying trial design and the assumptions held by the lay public, the solution is unlikely to be simple.

Some women reported that they would let mother Nature do her work. They were reluctant to actively choose for an intervention in an (perceived) uncomplicated pregnancy. Lyerly at all reported that women focused on risks associated with medical interventions during pregnancy, not taking into account the demonstrable risk to both woman and fetus of not intervening [23].

Many women were surprised to learn that the doctor does not always know what is best. This was also reported by Mohanna: ‘Some patients will prefer to assume that [*My*] doctor knows best [*about me and my baby*], and are not happy to enter into the discussion of uncertainty that a trial and the issue of informed consent will raise’ [24].

Counseling by research staff, instead of by the treating physician, seemed to influence participation positively, which has also been suggested in the review by Tooher and collegues [7].

To reduce feelings of uncertainty and stress, further research could elaborate on the work by Junghans and colleagues, where an opt-out design for low-risk interventions was proposed [25, 26]. This could not only increase participation rates, leading to a more representative study group, but might also shift the responsibility and difficult decision process from pregnant women to the health professionals. Ethical committees could or should be responsible for identifying low-risk trials eligible to run in this system. Patients could for example be informed about this general policy of opt-out consent when entering the hospital, invited to sign a general informed consent about the use of data to improve quality.

Alternatively, one could think of a trial risk classification system, where the potential risks of a trial are communicated with uniform labels, much like energy labels, to make them more transparent for patients. An “A” classification for example could mean that widely used interventions are compared, with no additional risk compared to usual clinical practice. A classification of “E” could mean that the new intervention being tested is highly experimental. One could imagine that health professionals generally recommend participation in all “A” trials, instead of explicitly leaving the choice to the patient.

Uncertainty could also be reduced, and awareness improved, if pregnant women became more familiar with scientific research in general, and studies in pregnancy specifically. This calls for a national public campaign. In 2008 the ‘Get Randomized’ campaign was launched in the UK, informing the public about the importance of clinical trials using television, radio and newspaper advertising. The campaign increased public awareness of clinical trials, but those who did recall the ads were not more inclined to personally take part in a clinical trials if invited, than those who did not remember [27]. In the United States a comparable campaign celebrating the ‘everyday medical heroes’ of clinical research has been established. Its initiators believe the public has a poor and often negative understanding of clinical research [28].

For trials in pregnancy, general information leaflets, available when entering a midwifery practice or a hospital, could introduce the goals, methods and necessity of scientific research. Health professionals could discuss trial participation in general, and trials in pregnancy in specific, early in a pregnancy. Or a national public campaign that raises awareness and reduces barriers to participation in trials could be considered. All this could habituate women more to scientific research and the methods used in it.

## Conclusion

We identified seven themes that influenced the decision to participate in a trial. Contributing to scientific research was for many participants their main motive for participation in the trial, while others were motivated because the intervention seemed favorable and was not available outside the trial. Key motives for non-participation were a negative feeling towards the intervention, either because it might do harm, or for practical reasons. We noted that uncertainty about scientific research or the intervention was of considerable importance.

Measures should be taken to habituate pregnant women more to scientific research and the methods used in it. Without pregnant womens’ contribution and participation, we would not be able to advance our understanding of the effectiveness of interventions in pregnancy and childbirth.

## Additional file

Additional file 1:
**Topic list to guide the interview.** (ZIP 13 kb)
