# Supplementary material for: Pregnant womens’ concerns when invited to a randomized trial: a qualitative case control study
Source: BMC Pregnancy Childbirth. 2015 Sep 4;15:207. doi: 10.1186/s12884-015-0641-x (PMC4560072; doi:10.1186/s12884-015-0641-x)
Supplement: Additional file 1: — Topic list to guide the interview. (ZIP 13 kb) [file 12884_2015_641_MOESM1_ESM.zip › 7222683031549698_add1.doc]

APPENDIX 1: TOPIC LIST

**First question:** What did you decide to/not to participate in the study?

**Topics:**

• *Personal benefit:* therapeutic benefit of participation, most recent therapy, satisfaction, more/better monitoring of the disease, better relation with the caregiver, freedom

• *Altruism:* contribution to science, please the caregiver, help other patients with this condition

• *Knowledge/information provision:* to know exactly where you choose for, to be able to stop at any moment, randomization, placebo, blinded research/double blinded research, objective of the study, patient information

• *Concern/ distrust:* negative effects of treatment, known treatment better, additional injections/medication, concern informed consent, fear for the unknown, loss of control, infringement of privacy, study design, stress

• *Organizational:* appointments, time for consideration, extra consultations/ injections, time and costs for travel, job, child care, time for “research consult”

• *Attitude*: attitude to scientific research

• *Sociale environment:* influence partner/ environment/internet, who decides, care provider not enthousiastic about study, obligation to caregiver, moment of counseling.
