# Supplementary material for: Pregnant womens’ concerns when invited to a randomized trial: a qualitative case control study
Source: BMC Pregnancy Childbirth. 2015 Sep 4;15:207. doi: 10.1186/s12884-015-0641-x (PMC4560072; doi:10.1186/s12884-015-0641-x)
Supplement: Additional file 1: — Topic list to guide the interview. (ZIP 13 kb) [file 12884_2015_641_MOESM1_ESM.zip › 7222683031549698_add2.doc]

APPENDIX 1: TOPIC LIST

**Openingsvraag:** Wat deed u besluiten om wel/niet mee te doen aan het onderzoek?

**Topics:**

• *Persoonlijk voordeel:* therapeutisch voordeel bij deelname, meest recente therapie,

voldoening, meer/betere monitoring van ziekte, betere relatie met behandelaar, vrijheid

• *Onbaatzuchtigheid:* bijdrage wetenschap, behandelaar een plezier doen, andere patiënten

met deze aandoening helpen

• *Kennis/informatieverstrekking:* precies weten waar je voor kiest, elk moment kunnen stoppen,

randomisatie, placebo, geblindeerd onderzoek/dubbel blind onderzoek, doel van de studie,

patiëntinformatie

• *Bezorgdheid/wantrouwen:* nadelige gevolgen behandeling, bekende behandeling beter,

(extra) injecties/medicatie, bezorgdheid informed consent, angst voor onbekende, verlies

controle, inbreuk privacy, studiedesign, stress

• *Organisatorisch:* afspraken, bedenktijd*,* extra consulten/injecties, reistijd en kosten, werk,

kinderopvang, tijd voor “onderzoeksconsult”

• *Attitude*: houding ten opzichte van wetenschappelijk onderzoek

• *Sociale omgeving:* invloed partner/omgeving/internet, wie maakte keuze, behandelaar niet

enthousiast over studie, verplichting ten opzichte van behandelaar, moment van counseling
